# Vaccination of Adult Patients with Systemic Lupus Erythematosus in Portugal

**DOI:** 10.1155/2016/2845617

**Published:** 2016-03-16

**Authors:** Maria Francisca Moraes-Fontes, Ana Margarida Antunes, Heidi Gruner, Nuno Riso

**Affiliations:** ^1^Unidade de Doenças Auto-Imunes, Serviço Medicina 7.2, Hospital Curry Cabral, Centro Hospitalar Lisboa Central, 1069-166 Lisbon, Portugal; ^2^Núcleo de Estudos de Doenças Autoimunes (NEDAI) da Sociedade Portuguesa de Medicina Interna, Portugal

## Abstract

In the wake of the Portuguese vaccination program 50th anniversary it seems appropriate to review vaccination in patients with systemic lupus erythematosus. Controversial issues as regards the association between autoimmune diseases, infections, and vaccines are discussed as well as vaccine safety and efficacy issues as regards chronic immunosuppressant (IS) drug therapy. After a brief overview of national policies, specific recommendations are made as regards vaccination for adult patients with SLE with a particular focus on current IS therapy and unmet needs.

## 1. Introduction

Vaccines induce long lasting protective immunity against microbial pathogens, prevent clinically relevant infections, and constitute one of the most important medical interventions ever. Nevertheless, vaccination plans differ across nations in several respects: they are not always free of charge nor compulsory, not all schools require a vaccination certificate, there are different brands and combinations, boosters are given at different times and in some areas of the world, and due to political interference, vaccination is actually banned [1]. This review describes the Portuguese National Vaccination Plan and provides the clinician with an easy guide to the expected vaccination status according to a patient's age. Vaccine side-effects with a focus on autoimmunity and specific indications for vaccination in SLE patients are discussed together with the expected impact of immunosuppression upon vaccine efficacy and safety.

## 2. The Portuguese National Vaccination Plan

The National Vaccination Plan (NVP) in Portugal commemorates 50 years in 2015. It was officially started in 1965 and is free of charge for all, including migrants. It has also been continuously updated by governmental decree up until June 2015, when pneumococcal conjugate vaccine to newborns was added [2]. Of note, there are also specific recommendations for unvaccinated adult immigrants [3]. The state also actively implements rubella vaccination to every nonpregnant woman aged over 18 whose vaccination status is unknown, even in the postpartum period. Only diphtheria and tetanus are compulsory in Portugal, with revaccination being recommended every 10 years. Nevertheless, vaccination denial is rarely reported [4], refusal is generally met with disapproval, and vaccination procedures are highly regulated. In addition, should patients lose their vaccination record, it may be recovered through a registration database used in every health centre since the year 2000 [5]. All nationally administered vaccines are imported.

Most of the information on current rates of infection is available from websites issued by the Portuguese Directorate General for Health. Diphtheria, poliomyelitis, rubella, and measles have been eradicated in Portugal [6] but there are still sporadic reports of rural tetanus in unvaccinated individuals [7], pertussis in the first year of life [8], and mumps [9]. Furthermore, there is still a notoriously high estimated incidence of hepatitis B (1.98/100000) [10] and tuberculosis (21.6/100000) [11], the latter remaining one of the highest in Western Europe. Accordingly, the latest NVP has been very recently updated [12].

In Portugal, depending on year of vaccine introduction and patient age, some patients are not expected to have been vaccinated (Table 1). For BCG, it is difficult to provide such an age estimate because there was a revaccination policy for Mantoux anergic individuals (the latter was stopped in 2005). Nevertheless, BCG vaccination at birth was implemented in 1963 and the typical vaccination scar may be recognized. Of note, hepatitis A vaccination is not part of the NVP but a recent Portuguese study showed that individuals under 25 years of age are at particular risk of infection [13].

## 3. The Etiological Role for Infections and Vaccination in Autoimmune Diseases

Vaccines are not without risk and the degree of vaccine-related risk should always be compared to that associated with the corresponding natural infection. The issue of an etiological role for infections and vaccination in autoimmune diseases (AID) remains controversial. Autoimmunity is generally assumed to result from complex interaction between genetic traits and environmental factors and therefore it may be argued that until the pathogenesis of AID is fully understood, the real risk of an infection or vaccination cannot be clearly defined. Furthermore, autoimmune responses are not necessarily accompanied by any clinical manifestation and may precede disease expression and their frequency is inversely related to a global decline in infections [14]. In addition, epidemiological studies are difficult, as humans accumulate many infections by the time early adulthood is reached and while infections are common, AID are much rarer diseases. Insomuch as several infections have been associated with the onset of AID, both experimentally and in humans [15], distinguishing temporal association from causality remains a daunting task, exemplified by the link between narcolepsy and the H1N1 strain of influenza epidemic described in Northern Europe [16], but not in the USA [17]. Vaccine-related AID has been convincingly demonstrated, an example of which is the Guillain-Barré syndrome specifically associated with the 1976-77 vaccination campaign using the A/New Jersey/8/76 swine-flu vaccine [18]. Other rarely described associations have been described (Table 2).

Measles infection itself is associated with autoimmune thrombocytopenia [19] and prior suspicions of risks for multiple sclerosis, type 1 diabetes, autism, and rheumatoid arthritis have been subsequently disproven (Table 3). Adjuvants licensed for human use are coadministered in all except live vaccines, to enhance immunogenicity [20], and mostly consist of aluminium phosphate/hydroxide, oil in water emulsions (squalene), and liposomes [21]. Aluminium in vaccines and diet has been convincingly shown to lack adverse effects in infants in the first year of life [22] but aluminium itself, squalene, and all other currently used adjuvants have been associated with autoimmunity in a recent collection of reports grouping gulf war syndrome, siliconosis, macrophagic myofasciitis, and postvaccination syndrome collectively known as ASIA, autoimmune/inflammatory syndrome. In ASIA, the causative role of adjuvants is based on experimental models for which several immune-mediated mechanisms have been proposed [23]. An attempt to establish causality has to be done on a case-by-case basis, through the demonstration of a consistent nonrandom temporal association between AID and vaccination. So far, there are no minimum specific criteria established for AID induced by vaccination diagnosis and studies are awaited to verify this hypothesis. Whether SLE drug therapies such as hydroxychloroquine are protective in this respect remains speculative.

## 4. Vaccination Status in SLE Patients according to the Portuguese National Vaccination Program

According to EULAR recommendations, vaccination status should be assessed in the initial workup of patients with autoimmune conditions [24]. It is expected to vary depending on the patient's age and corresponding NVP. In clinical practice one should bear in mind that SLE mostly affects women and that the mean age of diagnosis is 31 years [25]. With reference to Table 1, when the schedule of the Portuguese NVP is taken into consideration, the only vaccination that remains a real concern in Portuguese patients is that against human papilloma virus (HPV). In the general population, epidemiological studies document a significant risk of infection until the age of 50, such that sexually active women are thought to potentially benefit from vaccination [26, 27]. This benefit is expected to be even higher in patients with SLE due to higher rates of persistent HPV infection, high-grade cervical dysplasia, and cervical cancer [28–31]. Pertussis,* Haemophilus influenzae* type B, and Meningococcal C would generally only be expected to occur in infants but the latter two are of particular concern in splenectomised patients. As already mentioned, poliomyelitis, measles, and rubella have been eradicated in Portugal. In the light of the recent influx of patients into Europe, special attention should nevertheless be paid to the following vaccinations:* Haemophilus influenzae *type B,* Neisseria meningitidis*, rubella (for women of childbearing age), tetanus, and hepatitis A and B. It must be emphasized that vaccination should preferably be performed in patients with stable disease [24] and as a general rule, whenever possible, titres should be checked, infection excluded, and vaccination administered accordingly.

## 5. Recommended Vaccines in SLE Patients

There are no clinically applicable scores that allow for the prediction of individual risk of infections in patients with SLE, particularly in those patients on immunosuppressive (IS) drugs which generally increase the risk of infection. Infection is, at present, one of the leading causes of death in SLE patients [25, 32, 33]. However, most bacterial, viral, fungal, and other infections that occur in SLE patients, namely,* Escherichia coli*,* Staphylococcus aureus* [34], cytomegalovirus, parvovirus B19, herpes simplex and Epstein-Barr virus [35],* Cryptococcus neoformans*,* Candida albicans* [36], and* Pneumocystis* [37], are not preventable by vaccines, as these have as yet to be developed and childhood/adolescent immunization against these pathogens will be highly desirable once these become available. In stark contrast, there are effective vaccines against invasive* Streptococcus pneumoniae* infections, to which SLE patients are particularly prone [38] and which are therefore recommended. Other infections of epidemic/pandemic nature such as influenza cause significant morbidity and mortality in the general population and yearly influenza vaccination is also recommended in patients with SLE [39, 40]. These recommendations are put forward by expert panels and take into account the frequent use of IS medication despite a lack of evidence that SLE patients are more frequently or severely affected by influenza or present additional risks of bacterial superinfection due to corticosteroid use [41]. In Portugal, pneumococcal polysaccharide vaccine and annual influenza vaccines are optional and partially state subsidized while the pneumococcal conjugate vaccine is not state supported for adults. Optimally, both pneumococcal vaccines should be administered following specific administration timings recently reviewed in detail [42]. Vaccinations commonly used in adult Portuguese SLE patients are presented in Table 4.

## 6. The Effect of Immunosuppressive Drugs on Vaccine Efficacy and Safety

Drugs used in chronic immunosuppression of SLE patients have a multiplicity of targets, may deplete cellular subsets, target intracellular signals involved in B and T-cell activation, and inhibit macrophage function. Few studies have systematically addressed the possibility that the most commonly chronically used IS drugs affect the rates of seroconversion after influenza and pneumococcal vaccination and even fewer have studied the long term protective effect of vaccination.

SLE patients may have a blunted immune response to influenza vaccination [43]. Exceptionally, a single study evaluated the effect of hydroxychloroquine and a stable dose of prednisolone and azathioprine on the rate of seroconversion one month following influenza vaccination and found that only azathioprine was associated with lower influenza antibodies [44]. It is currently unknown whether SLE patients maintain their titres following vaccination allowing for sufficient protection during the whole influenza season. Although the effect of mycophenolate mofetil (MMF) and rituximab (RTX) has not specifically been assessed in lupus patients, MMF diminishes influenza seroconversion in transplant recipients [45] and RTX has a similar effect in rheumatoid arthritis (RA) patients [46, 47]. In the latter patients, methotrexate does not significantly diminish humoral responses to vaccination [48, 49].

SLE patients may display nonprotective responses to the pneumococcal vaccine but no correlation has been made with the type and dose of IS [50]. In SLE patients, hepatitis B vaccine response may be impaired and serological responses should be assessed after vaccination [51]. RTX reduces responses to pneumococcal vaccine but this has only been studied in RA [47, 52]. Ideally, patients should be vaccinated before undergoing B cell depletion therapy. Belimumab does not seem to reduce vaccine antibodies to influenza, pneumococcal, and tetanus vaccines [53]. As a general rule, inactivated vaccines should be administered at least 14 days before initiation of immunosuppressive therapy to optimize immunogenicity.

It should be noted that even though childhood chickenpox can be associated with severe complications [54] and the vaccine is safe and effective [54, 55], chickenpox immunization is only offered to children in Australia, Canada, Germany, Korea, Qatar, Saudi Arabia, Taiwan, Uruguay, Sicily, and Madrid [56]. The vaccine is live, may cause disseminated infection in immunocompromised patients [57], and effectively remains excluded from most vaccination programs as there are still widespread worries that it may increase the risk of chickenpox and shingles in older people. On the other hand, herpes zoster is still the most common viral infection in SLE patients treated with cyclophosphamide and mycophenolate mofetil [58]. There is a zoster vaccine which is 50 to 70% effective. It has been licensed since 2006 for immunization of immunocompetent individuals over age 60 years with no history of recent zoster [59, 60] and has also been shown to be effective between 50 and 59 years of age [61]. It cannot however be routinely recommended in SLE patients because it is a live vaccine which may pose a danger of generalized and even fatal infections in IS patients. It is not recommended in patients that test negative for varicella-specific antibodies, indicating absence of previous exposure. According to EULAR guidance, zoster vaccination is contraindicated when steroids are used for ≥14 days, at a dose of 20 mg of prednisone equivalent or higher and with the use of azathioprine ≥3.0 mg/kg/day or methotrexate at ≥0.4 mg/kg/week [24]. Results of a recent trial are awaited, intended to test safety, tolerability, and immunogenicity of the zoster vaccine in patients receiving chronic/maintenance corticosteroids [62]. No information on MMF or belimumab is available as regards zoster vaccination safety. As a general rule it seems prudent to check immune reconstitution in patients that have been subjected to IS therapy in high dosage or for long periods of time before zoster vaccination is prescribed. Live vaccines are contraindicated in patients undergoing biological therapy [42].

Detailed information about traveler needs and emerging infections [21] is beyond the scope of this paper. As already mentioned, live vaccines such as for yellow fever may cause an infection in significantly IS individuals. On the other hand, pathogen born yellow fever infection has a high mortality. Personalized decisions should therefore be made as regards vaccine administration, therapy delays, or drug holidays. Of note, yellow fever vaccine is safe in HIV positive individuals with adequate CD4^+^ T-cell counts [63] and in transplant recipients [64].

## 7. Conclusion

In conclusion, the overall balance is in favour of vaccination in patients with SLE. Infection/vaccine induced AID remains a theoretical possibility and there are documented effects of IS drugs on vaccine efficacy and safety. Vaccination records according to NVP should be routinely performed in the initial workup of SLE patients and additional vaccinations prescribed as required, bearing in mind that vaccination should be preferably performed when the disease is in remission. Seasonal influenza, pneumococcal, and HPV vaccination are actively encouraged. Whenever possible, protective titres should be checked for rubella and hepatitis A and B, preventing unnecessary vaccination. Live vaccination must be individualized and may be performed in non- or mildly immunosuppressed patients but data is awaited regarding their safety in patients that require higher dosages of IS. As a whole, vaccination is safe and should be supported as part of standard of care in patients with SLE in whom infections are a significant cause of morbidity and mortality. It is also hoped that new vaccines will greatly contribute to progress in improving healthcare and that old vaccines will be optimized to provide extended protection.

## Figures and Tables

**Table 1:** (a) Vaccination, (b) vaccination as part of NVP, (c) ages at which vaccination is administered, and (d) age above which patients are not expected to have been vaccinated in Portugal up to December 2015; m: months; y: years.

(a) Infection	(b) Vaccination as part of NVP	(c) Age of administration (m or y)	(d) Age (y) above which patients are not expected to be vaccinated
Pertussis	1963–1965	2 m	52
Poliomyelitis	1965	2 m	50
Mumps and rubella	1974	12 m	41
Measles	1987	12 m	44
Hepatitis B	2000 (0 + 13 years old at onset of campaign)	Birth	28
*Haemophilus influenzae* B	2000	2 m	28
Human papilloma virus	2008 (13 y), 2009 (17 y)	12 y	20–23
Meningococcal C	2006	12 m	10

**Table 2:** Reported associations between vaccination and autoimmune diseases.

Vaccine/adjuvant	Disease
Measles, mumps, and rubella	Immune thrombocytopenic purpura [19]
Hepatitis B vaccine 1991–1997	Multiple sclerosis [65], one study
H1N1 influenza vaccine in 2009	Narcolepsy [66]
Hepatitis B vaccination	Undifferentiated connective tissue disease [67, 68]
Human papilloma virus	Ovarian failure and lupus [69]

**Table 3:** Lack of associations between vaccination and autoimmune diseases.

Vaccine/adjuvant	Disease
Hepatitis B vaccine 1991–1997	Multiple sclerosis [70], 5 studies
*Haemophilus influenzae *type B	Type 1 diabetes mellitus [71]
Measles, mumps, and rubella/thimerosal	Autism, 20 studies [72]
Tetanus, influenza, and hepatitis B	Rheumatoid arthritis [73]

**Table 4:** Vaccine recommendations in adult Portuguese patients with systemic lupus erythematosus.

Diphtheria and tetanus	Every 10 years

Human papilloma virus	Sexually active females

Seasonal influenza	Yearly

Pneumococcal polysaccharide	Repeat once after 5 years [74]

Pneumococcal conjugate	Single administration [75]
